# Running Patterns in *LaLiga* Before and After Suspension of the Competition Due to COVID-19

**DOI:** 10.3389/fphys.2021.666593

**Published:** 2021-04-26

**Authors:** Diego Brito de Souza, Roberto López-Del Campo, Ricardo Resta, Victor Moreno-Perez, Juan Del Coso

**Affiliations:** ^1^Exercise Physiology Laboratory, Camilo José Cela University, Madrid, Spain; ^2^Department of Competitions and Mediacoach, LaLiga, Madrid, Spain; ^3^Sports Research Centre, Miguel Hernandez University of Elche, Alicante, Spain; ^4^Centre for Sports Studies, Rey Juan Carlos University, Fuenlabrada, Spain

**Keywords:** football, sports competition, elite athlete, professional athlete, sport performance

## Abstract

In the first wave of the COVID-19 outbreak (spring 2020), the first division of professional soccer in Spain (*LaLiga*) was suspended for 12 weeks as part of the lockdown imposed by the Spanish health authorities. Professional soccer players were confined to home for 8 weeks and then a retraining period of 4 weeks was set before the first competitive match. When competition was resumed, professional soccer teams competed in a congested calendar (11 matchdays in 39 days) while some in-game regulations were altered (up to 5 substitutions, refreshment pauses). The current research presents an analysis of running patterns before suspension and after resumption of *LaLiga* to determine how the lockdown affected players’ physical performance. To aid in this purpose, a pairwise comparison was performed of running patterns of the 2019–2020 vs. 2018–2019 season (i.e., control season). Using a two-way ANOVA (season x matchday), it was found that there was no main effect of the season on total running distance per match (*P* = 0.288) nor in the distances covered < 14.0 km/h (*P* = 0.294), at 21.0–23.9 km/h (*P* = 0.266), and at ≥ 24.0 km/h (*P* = 0.112). Only the distance at 14.0–20.9 km/h was affected by the season (*P* = 0.019) with a lower running distance on matchday 34 in the 2019–2020 vs. 2018–2019 season. The number of substitutions (from 2.9 to 4.5 substitutions per game; *P* < 0.001) and match duration (96 vs. 100 min; *P* < 0.001) significantly increased after resumption respect to the previous season. These data suggest that high-intensity running performance of professional soccer teams was maintained after the resumption of the competition while the alterations likely aided in the in-game regulations facilitated the maintenance of soccer physical performance.

## Introduction

During the spring of 2020, the outbreak of the coronavirus disease (COVID-19) caused the suspension of sports competition worldwide, as their suspension was one of the several actions taken by most countries to reduce the spread of the virus. In most European countries, suspension of sports competition was accompanied by the lockdown of territories and home confinement. In Spain, the first wave of COVID-19 impacted in March 2020 and national health authorities set a severe lockdown that entailed home confinement starting on March 14 (Castañeda-Babarro et al., 2020). At the beginning, the lockdown was set for a duration of two weeks and professional and elite athletes struggled to maintain their physical condition by training at home as it was believed that sports competitions would be resumed as soon as the lockdown finished (Sarto et al., 2020). However, at the end, home confinement lasted for 8 weeks and athletes had to perform multiple and innovative forms of home training in an attempt to mitigate the detraining effects of confinement on their physical conditioning.

In Spanish professional soccer, players tried to keep their training routines at their homes during home confinement, following individualized programs provided by the teams’ strength and conditioning staff. The training programs mainly included strength-based activities with body loads, proprioception activities, exercise performed with low range displacements and some endurance-based exercises such as running on a treadmill or cycling on a stationary bike (Barca Innovation Hub, 2020). Despite the effort of the teams’ staff, the inclusion of high-intensity running actions depended on the conditions of home confinement for each player. For this reason, the execution of soccer-specific displacements such as accelerations/decelerations, sprints, and changes of direction were difficult to perform at home for most players (Moreno-Pérez et al., 2020).

Due to the potential risks of infection and injury, most sports competition in Spain were not resumed after home confinement and they were concluded until the next season. However, the case of professional soccer was different to other sport competitions. Due to the economic revenues and the popularity of soccer, the suspension of the professional leagues was a matter of debate in health, social and sports forums. Most soccer governing bodies stood for the resumption of the competition to finish the championships after the lockdown was lifted, although there were calls to avoid an overly premature resumption of soccer competition in Spain and in other European countries (Corsini et al., 2020; Herrero-Gonzalez et al., 2020). In addition, there were statements that provided practical recommendations for the preparation of training sessions for professional soccerers when returning to competition after the lockdown (Bisciotti et al., 2020; Herrero-Gonzalez et al., 2020).

Spanish health and sports authorities set specific guidelines for the resumption of a few professional competitions (i.e., soccer, basketball). The guidelines for professional competition resumption were established keeping in mind athletes’ health status after the confinement, the reduction of the likelihood of COVID-19 infection during training and competition and the development of strategies for injury prevention (Herrero-Gonzalez et al., 2020). In fact, recent data suggest that professional soccer training and competition could have been carried out safely after the first wave of the COVID-19 pandemic using strict hygiene measures, regular PCR testing (Buldú et al., 2020; Meyer et al., 2020) and systematic contact tracing following confirmed cases (Carmody et al., 2020).

Specifically, for the first division of professional soccer in Spain (*LaLiga*), a retraining period of 4 weeks was established after lockdown and then the competition was resumed on June 8 2020 and the 11 fixtures left to finish the championship were successfully completed without any infections. As new waves of COVID-19 are impacting again in Spain and in other European countries, the analysis of the data of the previous season may be very useful in the case of future lockdowns that entail sports competition suspension and posterior resumption. In this regard, the need of investigating the effect of lockdown on soccer performance has been suggested by analysing running activity patterns and game statistics in the matches played after soccer competition resumed (Souza et al., 2020). To this regard, although soccer is a complex team sport in which success is based on the interaction of multiple physical, technical and tactical capacities of players and of team squads (Sarmento et al., 2018), the analysis of running performance after the competition resumption may be useful to understand the outcomes of the 2019–2020 season of *LaLiga*, as running activities during the matches are related to end-season ranking in a national league (Longo et al., 2019). In addition, the analysis of running performance after the competition resumption in the 2019–2020 season may be helpful to set specific guidelines, based on precedents, that aid the return to play after lockdowns. Hence, the aim of this article is providing a comparative analysis of match running performance in teams competing in *LaLiga* before and after the lockdown due to COVID-19.

## Methods

### Participants

The study sample was composed of 530 and 555 soccer players competing in *LaLiga* Santander for the 2018–2019 and 2019–2020 seasons, respectively. A total of 342 soccer players played on both seasons while the remaining 401 players only played on one of the two seasons under investigation. This sample corresponds to the entire population of professional soccer players that competed in *LaLiga* for these two seasons. The inclusion criteria were (a) being a soccer player competing in the first-division of soccer in Spain, (b) being professionally associated to one of the twenty teams competing in *LaLiga* and (c) playing at least one match in either the 2018–2019 and 2019–2020 seasons. In accordance with *La Liga*’s ethical guidelines, this investigation does not include information that identifies soccer players. The Institutional Review Board of the Camilo José Cela University approved this study, which is in accordance with the latest version of the Declaration of Helsinki.

### Experimental Procedures

This study is a descriptive and comparative analysis of match running performance in all teams competing in *LaLiga* in the 2018–2019 and 2019–2020 seasons. To aid in determining the effect of lockdown in soccer running performance, a pairwise comparison of running patterns was performed between these two seasons. The 2018–2019 season was established as a “control” season while the 2019–2020 season was considered as the “experimental” season because entailed normal competition for 27 matches, a suspension for12 weeks and resumption to finish the 11 fixtures remaining.

The analysis includes the average running distance per game for each of the 38 matchdays that compose the first division of professional soccer in Spain, for a total of 560 matches analysed (i.e., 380 matches per season). Data were obtained from *LaLiga*, which authorised the use of the variables included in this investigation. Data were extracted by a valid and reliable multicamera tracking system and associated software (Mediacoach^®^, Spain) that measures players’ running distance in total and at different speeds (i.e., below 14.0 km/h, between 14.0 and 20.9 km/h, between and 21.0 and 23.9 km/h and above 24.0 km/h). The number of running actions above 24.0 km/h was also obtained in each match to assess the number of sprints performed. Mediacoach^®^ records the position of each player on the pitch at 25 Hz using a stereo multi-camera system composed of two multi-camera units placed at either side of the midfield line. Each multi-camera unit contains three cameras with a resolution of 1920 × 1080 pixels which are synchronised to provide a stitched panoramic picture (Del Coso et al., 2020). The panoramic picture is then employed to create the stereoscopic view that allows triangulating all the players on the field to assess their position and to calculate running speed during the match. In the case of a lack of location of a player due to occlusions by another player, an experienced operator manually corrected the position during measurement. The validity of Mediacoach^®^ to assess running distance during soccer match play has been obtained through high agreement with the data obtained with Global Positioning System units (Felipe et al., 2019; Pons et al., 2019) and with data obtained from a reference camera system (i.e., VICON motion capture system (Linke et al., 2020)). Data on each variable was normalised as team’s running distance per match to obtain easier-to-use information for coaches and physical conditioning staff (Brito Souza et al., 2020). Additionally, the number of substitutions per match and match duration were also extracted to assess the effect of the in-game regulations introduced after the resumption of the competition.

### Statistical Analysis

Statistical analyses were carried out using the software IBM SPSS Statistics for Macintosh, Version 26.0 (IBM Corp., Armonk, NY, United States). Data were normally distributed in all variables as determined by the Shapiro-Wilk test. Additionally, the sphericity assumption was checked with Mauchly’s test. If this assumption presented a probability of *P* < 0.05, the Greenhouse-Geisser correction was used. To identify the effects of the lockdown on match running performance variables, we used a two-way analysis of variance (ANOVA) with within-between comparisons (season × matchday), and an LSD post-hoc analysis in those variables with a significant F test. To specifically examine the effect of lockdown on the fixtures performed after the resumption of the soccer competition, a sub-analysis comparing the last 11 fixtures (from the fixture 28 to the fixture 38) of the 2018–2019 and 2019–2020 seasons was performed. For this sub-analysis, we used unpaired t tests while the effect size was also calculated by using Cohen’s *d* units (Cohen, 1988). The significance level was set at *P* < 0.050.

## Results

**FIGURE 1:**
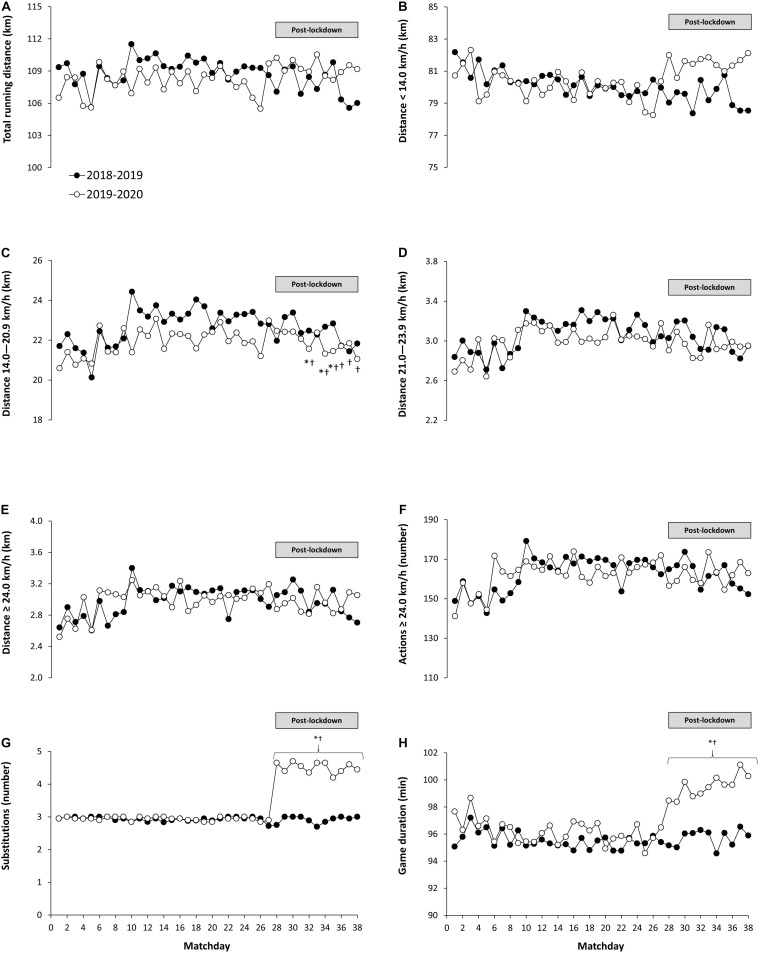
Total running distance per match, distance at different speed thresholds, number of sprints, players’ substitutions, and game duration in *LaLiga* in the 2018–2019 and 2019–2020 seasons. **(A)** Total running distance, **(B)** running distance covered at <14.0 km/h, **(C)** running distance covered between 14.0 and 20.9 km/h, **(D)** running distance covered between 21.0 and 23.9 km/h, **(E)** running distance covered at ≥24.0 km/h, **(F)** number of sprints covered at ≥24 km/h, **(G)** number of players’ substitutions, **(H)** game duration. Each dot represents mean value for 20 teams on each matchday for each season (*) Different from the same matchday in the 2018–2019 season, *P* < 0.05. (†) Different from matchday 27 in the 2019–2020 season, *P* < 0.05. Note: In the 2019–2020 season, the competition was suspended after matchday 27 due to the COVID-19 pandemic. The competition was resumed after 12 weeks (8 weeks of lockdown and 4 weeks of retraining) to complete the 38 matchdays that comprised *LaLiga.*

The two-way ANOVA revealed that there was no main effect of the season or season × matchday interaction on total running distance per match, in the distances covered < 14.0 km/h, in the distance covered between 21.0 and 23.9 km/h, and in the distance covered at ≥ 24.0 km/h (Figure 1; see Table 1 for F and *P* values). Likewise, there was no main effect of the season nor season × matchday interaction in the number of sprints performed at ≥ 24.0 km/h. However, there was a main effect of the season on the distance covered at 14.0–20.9 km/h (*P* = 0.019) with the post-hoc analysis revealing lower distances in the 2019–2020 season vs. 2018–2019 season for matchdays 32, 34, and 35 (*P* < 0.050). Additionally, the distance covered at 14.0–20.9 km/h was lower on matchdays 32, 34, 35, 36, 37, and 37 with respect to matchday 27 of the 2019–2020 season (*P* < 0.050).

**TABLE 1:** Main effects (season × matchday) and interaction in running patterns of professional soccer teams in *LaLiga* when comparing the 2018–2019 and 2019–2020 seasons.

**Variable**	**Season**	**Matchday**	**Interaction**
Total running distance	F = 1.361 *P* = 0.288	F = 1.405 *P* = 0.254	F = 1.588 *P* = 0.193
Distance at <14.0 km/h	F = 1.321 *P* = 0.294	F = 0.751 *P* = 0.415	F = 0.893 *P* = 0.433
Distance at 14.0–20.9 km/h	F = 11.657 *P* = 0.019	F = 1.553 *P* = 0.234	F = 1.037 *P* = 0.414
Distance at 21.0–23.9 km/h	F = 1.564 *P* = 0.266	F = 2.079 *P* = 0.128	F = 1.588 *P* = 0.193
Distance at ≥24.0 km/h	F = 3.470 *P* = 0.112	F = 1.305 *P* = 0.294	F = 1.482 *P* = 0.220
Actions at ≥24.0 km/h	F = 2.077 *P* = 0.209	F = 0.981 *P* = 0.436	F = 1.151 *P* = 0.361
Number of substitutions	F = 308.197 *P* < 0.001	F = 19.603 *P* < 0.001	F = 22.890 *P* < 0.001
Match duration	F = 7.200 *P* < 0.001	F = 3.522 *P* = 0.385	F = 7.344 *P* = 0.110

*In the 2019–2020 season, the competition was suspended at matchday 27 due to the COVID-19 pandemic. The competition was resumed after 12 weeks (8 weeks of lockdown and 4 weeks of retraining) to complete the 38 matchdays that comprised LaLiga.*

The two-way ANOVA also revealed main effects of the season, matchday and an interaction between these two factors in the number of players’ substitutions that the teams used per match (Table 1). Specifically, the number of substitutions was higher in all pairwise comparisons between the 2018–2019 vs. 2019–2020 season from matchday 28 to matchday 38 (Figure 1; *P* < 0.050). Furthermore, the number of substitutions was higher from matchday 28 to matchday 38 when compared to matchday 27 of the 2019–2020 (*P* < 0.050). There was also a main effect of the season on match duration (Table 1), indicating that match duration was higher from matchday 28 to matchday 38 in the 2019–2020 season with respect to the previous season (*P* < 0.050) while match duration was higher from matchday 28 to matchday 38 when compared to matchday 27 within the 2019–2020 season (*P* < 0.05).

In the sub-analysis of the last 11 matchdays of each season, total running distance and the distance at < 14.0 km/h were higher in the 2019–2020 season when compared to the 2018–2019 season (*P* < 0.050; Table 2). Additionally, the number of substitutions and match duration was higher the 2019–2020 season when compared to the 2018–2019 season (*P* < 0.050). On the contrary, the distance covered between 14.0 and 20.9 km/h was lower in the 2019–2020 season when compared to the 2018–2019 season (*P* = 0.034). There were no other differences between seasons in the remaining running performance variables in the last 11 matchdays of the seasons under investigations.

**TABLE 2:** Averaged running patterns of professional soccer teams in *LaLiga* in the last 11 fixtures of the 2018–2019 and 2019–2020 seasons.

**Variable**	**2018–2019**	**2019–2020**	***P* value**	**Effect size**
Total running distance	107.7 ± 1.5	109.3 ± 0.7	0.015	1.10
Distance at <14.0 km/h	79.3 ± 0.8	81.5.3 ± 0.4	<0.001	2.75
Distance at 14.0–20.9 km/h	22.4 ± 0.6	21.9 ± 0.5	0.034	−0.81
Distance at 21.0–23.9 km/h	3.0 ± 0.1	3.0 ± 0.1	0.226	0.10
Distance at ≥24.0 km/h	3.0 ± 0.2	2.9 ± 0.1	0.759	−0.13
Actions at ≥24.0 km/h	162.1 ± 6.6	162.1 ± 5.6	0.994	0.00
Number of substitutions	2.9 ± 0.1	4.5 ± 0.2	<0.001	14.7
Match duration	95.7 ± 0.6	99.5 ± 0.8	<0.001	6.6

## Discussion

This analysis reveals that, despite the lockdown imposed by the Spanish health authorities during spring 2020 to control the first wave of the COVID-19 pandemic, running performance in the professional soccer teams of *LaLiga* was well preserved after the resumption of the competition, which took place after 12 weeks of competition suspension. This maintenance of overall running performance during the match was evident when comparing data of the season disrupted by the pandemic (2019–2020) to a control season (2018–2019), as running performance in all speed thresholds -except for some matchdays in the distance covered at between 14.0–20.9 km/h (Figure 1C) were maintained or even increased. The sub-analysis of the last 11 fixtures revealed that, both, the total running distance, and the distance covered at low running velocity (i.e., <14 km/h) were increased in the 2019–2020 season (Table 2) respect to the previous season. This increase in total running distance and in low-intensity running were likely facilitated by the longer match duration and the possibility of reaching up to five substitutions per match. Interestingly, the running distance at above 21 km/h, which represents the running actions more associated to soccer performance, particularly when in possession of the ball (Brito Souza et al., 2020) match, were well preserved in the 2019–2020 in comparison to the control season. It is probable that the maintenance of running distance at high speed and the number of sprints per match after the resumption of the competition was associated to the lower distance covered at 14.0–20.9 km/h (Figure 1C and Table 2). Although it remains as a speculation, the reduction of running activities of moderate intensity (i.e., 14.0–20.9 km/h) may represent an enhanced pacing strategy during matches to ultimately maintaining high-intensity running (i.e., > 21 km/h) despite a potential lower physical condition due to home confinement and the congested calendar. Collectively, all this information suggests that high-intensity running performance of professional soccer teams in *LaLiga*, was maintained after the resumption of the competition in the 2019–2020 seasons despite the competition was suspended for 14 weeks, including 8 weeks of severe home confinement.

These outcomes of the current investigation were likely assisted by management and regulations that Spanish health and soccer authorities established for professional soccer after the lockdown. In fact, it was predicted that, when resuming soccer competition after the lockdown, professional players of *LaLiga* would experience physical challenges similar to the ones they usually undergo during the first official matches of the season (i.e., a progressive increase in running performance during the first official matches (Souza et al., 2020)) because the lockdown was long enough to expect detraining effects (Pereira et al., 2020). This scenario was predicted with the data at that time which indicated muscle weakness induced by the lockdown (Moreno-Pérez et al., 2020) despite staff and soccer players trying to maintain their soccer-specific physical condition by training at home. However, this potential scenario did not materialise because the Spanish soccer authorities ensured players’ health and safety and established regulations that avoided excessive fatigue while aiding soccer performance (Herrero-Gonzalez et al., 2020).

First, a retraining period of at least 4 weeks was set from the end of home confinement to the first competitive match. In this time, professional teams prepared their return to play following the recommendations of the Spanish Sports Council, in agreement with the Royal Spanish Soccer Federation (RSFF) and *LaLiga*, which established regulations to allow individual-only exercise routines for the first week of retraining with a progressive inclusion of small-group exercises until completing team trainings and 11-per side match simulation routines in the last weeks of the retraining period. Second, a minimum period of 72 h was set between matches as lower between-game recovery periods may entail accumulated fatigue and stress (Mohr et al., 2016) and could potentially lead to higher injury incidence (Bengtsson et al., 2014). Interestingly, the running patterns after the lockdown were preserved despite the teams completing the 11 matchdays remaining to finish the 2019–2020 season in ∼39 days (i.e., one game every 3.5 days). Of note, the previous year, the last 11 matchdays were completed in 63 days (i.e., one game every 5.7 days).

In the opinion of these authors, the specific modifications of the in-game regulations allowed after the lockdown were also key to maintaining players’ physical running patterns (especially those above 21 km/h) and hence, the integrity of the competition. The RSFF and *LaLiga* agreed to permit two extra players’ substitutions (for a total of up to 5 substitutions per match) although teams had to request substitutions in only three turns. The current data indicate that most teams used this in-game allowance as the mean number of substitutions in the last 11 fixtures of the 2018–2019 season was 2.9 substitutions per game and it reached 4.5 substitutions per game in the 2019–2020 season (Table 2). Habitually, substitutes cover greater running distances than players who complete the entire match (Bradley et al., 2014) which points toward a favourable outcome of the allowance of up to 5 substitutions to preserve running performance after the lockdown. In this regard, some authors have recently proposed keeping the increase in substitutions from three to up to five permanently, with the aim of mitigating overall soccer physical demands (Mota et al., 2020). Interestingly, the time chosen for the first substitution did not vary after the lockdown (58 ± 3 min in 2018–2019 and 57 ± 2 min in 2019–2020 for the las 11 matchdays) and the time played by substitutes was similar (25 ± 2 min in 2018–2019 and 26 ± 1 min in 2019–2020 for the las 11 matchdays) suggesting that team managers do not anticipate substitutions despite possessing two more substitutions than before.

Additionally, a mandatory use of refreshment pauses at minute 30 and 75 of each match was established to allow enhanced in-game recovery as the game was stopped for ∼2 min in each half. As a result, game duration increased from 96 min in the last 11 fixtures of the 2018–2019 season to 100 min for the same period of the 2019–2020 season (Table 1). This likely produced that total running distance and distance at < 14 km/h were higher in the 2019–2020 vs 2018–2019 season (Table 2), as players usually moved at a low intensity running to the side-line for refreshment. To compensate for the time used for these pauses, referees increased game duration in each half as reflected in the current investigation, but the effective time of play was probably conserved. To date, there is no data to determine how effective these refreshment pauses are to help players’ for in-game recovery, but the maintenance of the distance run at ≥ 21 km/h and the similar number of actions above 24 km/h in the post-lockdown period suggests that these drink breaks were be helpful to maintain running performance despite the congested calendar of the last 11 fixtures of the 2019–2020 season.

The current analysis describes an unusual situation produced by a virus pandemic and provides data on how health and soccer governing bodies were right about the proposition of new in-game regulations and by setting an appropriate time for retraining phase after home confinement. However, the analysis presents some limitations. First, the training routines performed at home during confinement and during the retraining period of 4 weeks were different between players and between teams. With the current data, we are unable to determine what teams selected the most optimal retraining strategies to maintain running performance after the resumption of competition. Additionally, the current analysis does not establish if those soccer teams that used a higher number of substitutions per game -up to five- were more able to maintain running performance after the resumption of competition. Last, the current analysis does not contain information about players’ internal load during the matches or an evaluation of wellness before the matches. This information could have been useful to determine if players felt more fatigue before matches due to the congested calendar set for the resumption of the competition or if they presented a higher internal load in comparison to the 2018–2019 season. Despite these limitations, the authors of this brief report honestly believe that the information provided here can be useful for coaches and strength and conditioning staff to understand how running performance during soccer competition can be maintained after a detraining period induced by home confinement or other analogue measures.

In summary, running patterns in professional soccer teams competing in 2019–2020 *LaLiga* were maintained when the competition was resumed after lockdown due to the first wave of COVID-19, especially the distance covered at > 21 km/h. The 11 matchdays left to finish the championship were played with ∼3.5 days of recovery between matches but establishing 4 weeks of retraining, the authorisation for up to 5 player’s substitutions during each match, and the mandatory use of refreshment pauses likely aided maintaining match running performance, at least when compared to that of the previous season. As the new wave of the COVID-19 pandemic are hitting most countries worldwide, it is expected that some sports competitions will have to be suspended to reduce the spread of the SARS-CoV2. The current data may be useful for sport’s governing bodies to value the use of unusual regulations to reduce the stress of sports, particularly in those circumstances where athletes have been confined to home or when the competition has been suspended for several weeks. As the duration of lockdown and sports competition interruption may be substantially different among countries, future investigations should determine the best guidelines for sports competition resumption after suspension due to COVID-19. These best guidelines should include information about the length of retraining and modulation of some in-game regulations.

## Data Availability Statement

The datasets generated for this study are available on request to the corresponding author. LaLiga is the owner of these data and this institution should approve any use of the data for further investigations.

## Ethics Statement

The studies involving human participants were reviewed and approved by Camilo José Cela University Ethics Committee. Written informed consent for participation was not required for this study in accordance with the national legislation and the institutional requirements.

## Author Contributions

All the authors have equally contributed to the conception and preparation of this investigation. All authors have read and approved the final version of the manuscript and agreed with the order of presentation of the authors.

## Conflict of Interest

RL-D and RR were LaLiga employees during the preparation of this work. The remaining authors declare that the research was conducted in the absence of any commercial or financial relationships that could be construed as a potential conflict of interest.

## References

[B1] Barca Innovation Hub (2020). *COVID-19: How Fc Barcelona Trains During The Isolation Period. Barca Innov Hub.* Available online at: https://barcainnovationhub.com/covid-19-how-fc-barcelona-trains-during-the-isolation-period/ (accessed March 24, 2021).

[B2] Bengtsson H. Ekstrand J. Waldén M. Hägglund M. (2014). Muscle injury rates in professional soccer increase with fixture congestion: an 11-year follow-up of the UEFA Champions League injury study. *Br. J. Sports Med.* 48 566–567. 10.1136/bjsports-2014-093494.19 23851296

[B3] Bisciotti G. N. Eirale C. Corsini A. Baudot C. Saillant G. Chalabi H. (2020). Return to soccer training and competition after lockdown caused by the COVID-19 pandemic: medical recommendations. *Biol. Sport* 37 313–319. 10.5114/biolsport.2020.96700 32879554 PMC7433324

[B4] Bradley P. S. Lago-Peñas C. Rey E. (2014). Evaluation of the match performances of substitution players in elite soccer. *Int. J. Sports Physiol. Perform.* 9 415–424. 10.1123/IJSPP.2013-0304 24413912

[B5] Brito Souza D. López-Del Campo R. Blanco-Pita H. Resta R. Del Coso J. (2020). Association of match running performance with and without ball possession to soccer performance. *Int. J. Perform. Anal. Sport* 20 483–494. 10.1080/24748668.2020.1762279

[B6] Buldú J. M. Antequera D. R. Aguirre J. (2020). The resumption of sports competitions after COVID-19 lockdown: the case of the Spanish soccer league. *Chaos Solitons Fractals* 138:109964. 10.1016/j.chaos.2020.109964 32518475 PMC7269962

[B7] Carmody S. Ahmad I. Gouttebarge V. Malhotra A. Glover D. Massey A. (2020). Infographics. Soccer-specific strategies to reduce COVID-19 transmission. *Br. J. Sports Med.* 54 1362–1364. 10.1136/bjsports-2020-102693 32788295

[B8] Castañeda-Babarro A. Coca A. Arbillaga-Etxarri A. Gutiérrez-Santamaría B. (2020). Physical activity change during COVID-19 confinement. *Int. J. Environ. Res. Public Health* 17 1–10. 10.3390/ijerph17186878 32967091 PMC7558959

[B9] Cohen J. (1988). *Statistical Power Analysis for The Behavioral Sciences*, Second Edn. Mahwah, NJ: Lawrence Erlbaum Associates.

[B10] Corsini A. Bisciotti G. N. Eirale C. Volpi P. (2020). Soccer cannot restart soon during the COVID-19 emergency! A critical perspective from the Italian experience and a call for action. *Br. J. Sports Med.* 54 1186–1187. 10.1136/bjsports-2020-102306 32209554

[B11] Del Coso J. de Souza D. B. Moreno-Perez V. Buldú J. M. Nevado F. Resta R. (2020). Influence of players’ maximum running speed on the team’s ranking position at the end of the Spanish Laliga. *Int. J. Environ. Res. Public Health* 17 1–11. 10.3390/ijerph17238815 33261014 PMC7729782

[B12] Felipe J. L. Garcia-Unanue J. Viejo-Romero D. Navandar A. Sánchez-Sánchez J. (2019). Validation of a video-based performance analysis system (Mediacoach§) to analyze the physical demands during matches in LaLiga. *Sensors (Switzerland)* 19:4113. 10.3390/s19194113 31547591 PMC6806213

[B13] Herrero-Gonzalez H. Martín-Acero R. Del Coso J. Lalín-Novoa C. Pol R. Martín-Escudero P. (2020). Position statement of the Royal Spanish Soccer Federation for the resumption of soccer activities after the COVID-19 pandemic (June 2020). *Br. J. Sports Med.* 54 1133–1134. 10.1136/bjsports-2020-102640 32546560 PMC7513256

[B14] Linke D. Link D. Lames M. (2020). Soccer-specific validity of TRACAB’s optical video tracking systems. *PLoS One* 15:e0230179. 10.1371/journal.pone.0230179 32155220 PMC7064167

[B15] Longo U. G. Sofi F. Candela V. Dinu M. Cimmino M. Massaroni C. (2019). Performance activities and match outcomes of professional soccer teams during the 2016/2017 Serie A season. *Medicina (Kaunas)* 55:496. 10.3390/medicina55080469 31408996 PMC6723654

[B16] Meyer T. MacK D. Donde K. Harzer O. Krutsch W. Rössler A. (2020). Successful return to professional men’s soccer (soccer) competition after the COVID-19 shutdown: a cohort study in the German Bundesliga. *Br. J. Sports Med.* 55 1–6. 10.1136/bjsports-2020-103150 32972979 PMC7788215

[B17] Mohr M. Draganidis D. Chatzinikolaou A. Barbero-Álvarez J. C. Castagna C. Douroudos I. (2016). Muscle damage, inflammatory, immune and performance responses to three soccer games in 1 week in competitive male players. *Eur. J. Appl. Physiol.* 116 179–193. 10.1007/s00421-015-3245-2 26377004

[B18] Moreno-Pérez V. Del Coso J. Romero-Rodríguez D. Marcé-Hernández L. Peñaranda M. Madruga-Parera M. (2020). Effects of home confinement due to COVID-19 pandemic on eccentric hamstring muscle strength in soccer players. *Scand. J. Med. Sci. Sport* 30 2010–2012. 10.1111/sms.13768 33463766

[B19] Mota G. R. Dos Santos I. A. Arriel R. A. Marocolo M. (2020). Is it high time to increase elite soccer substitutions permanently? *Int. J. Environ. Res. Public Health* 17 1–13. 10.3390/ijerph17197008 32992687 PMC7579365

[B20] Pereira L. A. Freitas T. T. Pivetti B. Alcaraz P. E. Jeffreys I. Loturco I. (2020). Short-Term detraining does not impair strength, speed, and power performance in elite young soccer players. *Sports* 8:141. 10.3390/sports8110141 33113840 PMC7692758

[B21] Pons E. García-Calvo T. Resta R. Blanco H. López Del Campo R. Díaz García J. (2019). A comparison of a GPS device and a multi-camera video technology during official soccer matches: agreement between systems. *PLoS One* 14:e0220729. 10.1371/journal.pone.0220729 31393932 PMC6687125

[B22] Sarmento H. Anguera M. T. Pereira A. Araújo D. (2018). Talent identification and development in male soccer: a systematic review. *Sport Med.* 48 907–931. 10.1007/s40279-017-0851-7 29299878

[B23] Sarto F. Impellizzeri F. M. Spörri J. Porcelli S. Olmo J. Requena B. (2020). Impact of potential physiological changes due to COVID-19 Home confinement on athlete health protection in elite sports: a call for awareness in sports programming. *Sport Med.* 50 1417–1419. 10.1007/s40279-020-01297-6 32468329 PMC7254973

[B24] Souza D. B. de González-García J. Campo R. L.-D. Resta R. Buldú J. M. Wilk M. (2020). Players’ physical performance in LaLiga when the competition resumes after COVID-19: insights from previous seasons. *Biol. Sport* 37 2–7. 10.5114/biolsport.2020.96856 33795911 PMC7996388

